# Unexpected origins: Bronchopulmonary dysplasia and the unusual spectrum of cystic lung disease

**DOI:** 10.5339/qmj.2024.qitc.27

**Published:** 2024-03-25

**Authors:** Irfan Ul Haq, Mansoor Ali Hameed, Shakeel Ahmed, Mushtaq Ahmad, Ibrahim Rasheed

**Affiliations:** 1 Pulmonary Medicine Department, Hamad General Hospital, Doha, Qatar Email: ihaq@hamad.qa; 2 Pulmonary Medicine Department, Al Wakra Hospital, Al Wakra, Qatar

**Keywords:** Lung cyst, asthma, bronchopulmonary dysplasia

## Introduction

Cystic lung diseases are a group of diseases that have a variety of presentations, but all have radiological characteristics of multiple air-filled cysts. Chest CT scans may incidentally reveal lung cysts. However, additional testing is often required to determine the underlying cause.

## Case Presentation

**Figure 1.**
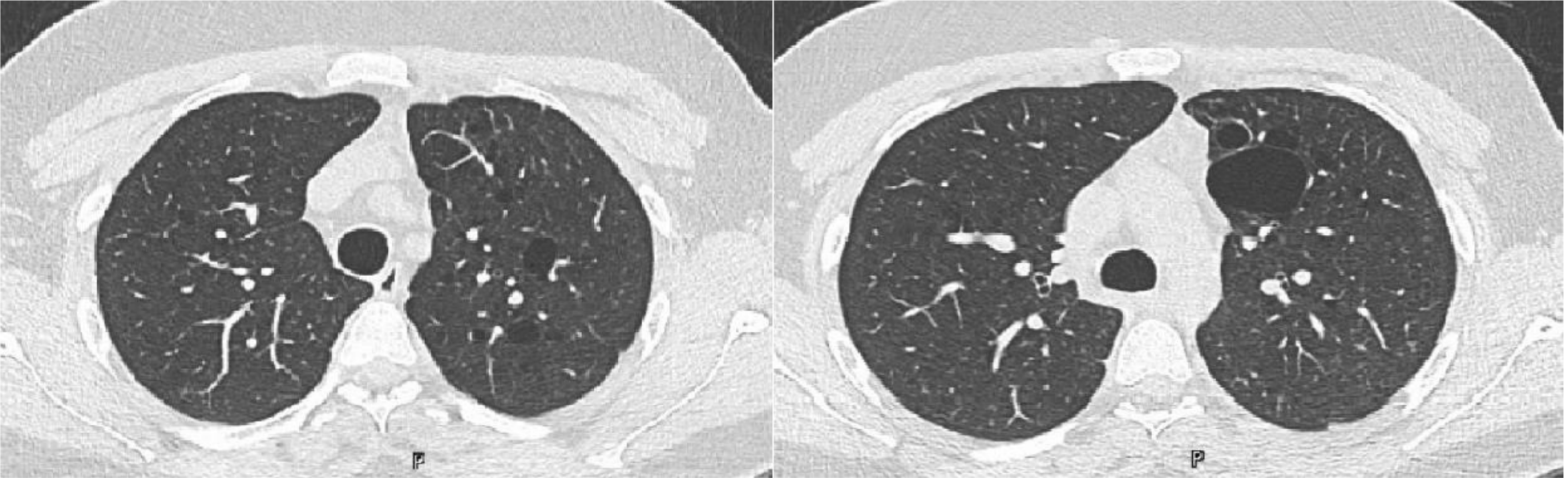
CT chest image showing bilateral cystic lung changes, with the left lung showing more prominent cysts.

A 25-year-old male was sent by the military to rule out asthma. He occasionally experienced wheezing and shortness of breath after mild exercise. He reported that he was prescribed inhalers for possible asthma when he was young. He had no other medical conditions, did not use illicit drugs, and did not smoke. He had no family history of lung conditions, including asthma. Upon reviewing his medical records, an abnormal chest CT scan from 10 years ago was discovered. Multiple lung cysts were seen on chest CT at the time, and this was confirmed by a follow-up scan that revealed no progression of the findings (Figure 1). Additional information from his online medical records indicated that he was treated for bronchopulmonary dysplasia and was born before term at 28 weeks. Bilateral cystic changes were noted on chest X-ray at three months of age. He then underwent spirometry and a methacholine challenge test, which was positive at PC 20 of 7.91 mg/ml. He was prescribed inhalers, and no additional testing was mandated.

## Discussion

Lymphangioleiomyomatosis, pulmonary Langerhans cell histiocytosis, Birt–Hogg–Dubé syndrome, and lymphoid interstitial pneumonia are the four most common causes of cystic lung disease.^1,2^ However, it is important to consider rare congenital causes of cystic lung disease, such as bronchopulmonary dysplasia, congenital lobar emphysema, congenital bulla, and bronchial atresia, particularly in young individuals who are born preterm.^3^ A thorough clinical history and evaluation of previous medical records can occasionally help narrow the differential diagnosis of cystic lung disease.

## Conflict of Interest

Authors declare they have no conflict of interest.
